# Associations of Gla-rich protein and interleukin-1β with coronary artery calcification risk in patients with suspected coronary artery disease

**DOI:** 10.3389/fendo.2025.1504346

**Published:** 2025-04-02

**Authors:** Cheng Zengwei, Gao Shiyi, Kang Pinfang, Gao Dasheng, Wang Jun, Hu Sigan

**Affiliations:** ^1^ Department of Cardiology, The First Affiliated Hospital of Bengbu Medical University, Bengbu, China; ^2^ Department of Cardiology, Wuhe County People’s Hospital, Bengbu, China

**Keywords:** coronary artery disease, coronary artery calcium score, Gla-rich protein, interleukin-1β, atherosclerosis

## Abstract

**Background:**

Gla-rich protein (GRP) and interleukin-1β (IL-1β) are recognized as reliable biomarkers for evaluating inflammation and are effective predictors of cardiovascular disease. However, the relationship between GRP, IL-1β, and coronary artery calcification (CAC) in patients with suspected coronary artery disease (CAD) remains unclear. Therefore, we investigated the association between these inflammatory biomarkers (GRP and IL-1β) and CAC in patients with suspected CAD.

**Methods:**

This prospective study included patients with suspected CAD who underwent coronary computed tomography angiography (CTA). Fasting venous blood samples were collected at admission, and GRP and IL-1β levels were quantified using enzyme-linked immunosorbent assays (ELISA). The Agatston score was calculated to assess coronary artery calcification (CAC) based on coronary CTA findings.

**Results:**

A total of 120 patients were included in this study. Multivariate logistic regression analysis revealed that GRP [odds ratio (OR), 1.202; 95% confidence interval (CI), 1.065-1.356; *p* = 0.003] and IL-1β (OR, 1.011; 95% CI, 1.002-1.020; *p* = 0.015) were independent risk factors for CAC severity. Receiver operating characteristic (ROC) curve analysis demonstrated that GRP had a predictive ability for CAC, with an area under the curve (AUC) of 0.830 [95% CI (0.755, 0.904)]. IL-1β exhibited an AUC of 0.753 [95% CI (0.660, 0.847)]. The combination of GRP and IL-1β in a predictive model improved the AUC to 0.835. Additionally, GRP and IL-1β levels showed a strong positive correlation (*r* = 0.6861, *p* < 0.05), and GRP was significantly associated with CAC severity (*r* = 0.5018, *p* < 0.05).

**Conclusions:**

Elevated levels of GRP and IL-1β, as inflammatory biomarkers, were associated with CAC in patients with suspected CAD. These biomarkers may provide valuable insights into the pathophysiology of coronary artery calcification and contribute to improved risk stratification in this patient population.

## Introduction

Atherosclerotic cardiovascular disease (ASCVD), including coronary artery disease (CAD), remains a leading global cause of mortality, accounting for over 30% of annual deaths worldwide. ASCVD also poses a substantial health burden on a global scale (1). From 1990 to 2019, the prevalence of ASCVD increased significantly, from 271 million to 523 million cases, with a concurrent rise in fatalities from 12.1 million to 18.6 million over the same period (2). Notably, the majority of CAD-related deaths occur outside healthcare settings, with nearly half being sudden (3).

Coronary artery calcification (CAC), as detected by coronary computed tomography angiography (CTA), is a well-established predictor of adverse outcomes in CAD patients and is positively correlated with an increased risk of myocardial infarction (4). As the severity of CAC intensifies, the likelihood of myocardial infarction rises correspondingly. Moreover, the extent and location of CAC play crucial roles in determining prognosis. Despite these associations, routine screening of the general population for CAC using coronary CTA is discouraged due to concerns regarding radiation exposure, the risk of contrast-induced nephropathy, and associated financial costs.

Emerging clinical evidence highlights the independent predictive value of inflammatory biomarkers for future cardiovascular events (5). Gla-rich protein (GRP), a novel member of the vitamin K-dependent protein (VKDP) family (6), has been recognized for its dual role in inhibiting pathological calcification and exerting anti-inflammatory effects in both joint and cardiovascular conditions (7, 8). Similarly, interleukin-1β (IL-1β), a potent pro-inflammatory cytokine, plays a pivotal role in arterial calcification, particularly within the context of atherosclerosis (9, 10).

Based on this, we hypothesized that serum levels of GRP and IL-1β might be associated with CAC risk in patients with suspected CAD, offering potential insights into early detection and therapeutic strategies targeting CAC in this specific population.

## Patients and methods

### Study population and design

This single-center observational study consecutively enrolled patients with a low to intermediate pretest probability of CAD, who were admitted to the First Affiliated Hospital of Bengbu Medical University between August 2022 and April 2023 and underwent coronary CTA. Patients were excluded if they had liver or kidney dysfunction, acute or chronic infections, malignancies, hematological disorders, immune system diseases, abnormal calcium metabolism, or severe osteoporosis.

This study followed the principles outlined in the Declaration of Helsinki and received approval from the First Affiliated Hospital of Bengbu Medical University Ethics Committee (approval number: 2023YJS287). All subjects signed informed consent.

### Blood sampling

Upon admission, laboratory tests were performed to measure fasting serum levels of total cholesterol, triglycerides, high-density lipoprotein cholesterol (HDL-C), low-density lipoprotein cholesterol (LDL-C), lipoprotein(a), calcium, phosphorus, albumin, creatinine, C-reactive protein (CRP), alkaline phosphatase, blood urea nitrogen (BUN), neutrophil count, lymphocyte count, hemoglobin, and platelet count. Additionally, the calcium-phosphorus product, neutrophil-to-lymphocyte ratio (NLR), and platelet-to-lymphocyte ratio (PLR) were calculated.

A total of 4 mL of venous blood was collected into an anticoagulant tube post-admission. The samples were then centrifuged at 3000 rpm to separate and preserve the serum, which was stored at -80°C for future analyses. Serum levels of GRP and IL-1β were quantified using enzyme-linked immunosorbent assay (ELISA) kits obtained from Shanghai Youxuan Biotechnology Co., Ltd., following the manufacturer’s protocols.

### Coronary artery calcium score

Coronary CTA examinations were performed on all patients using a 256-slice spiral CT scanner at the CT unit of the First Affiliated Hospital of Bengbu Medical University. The resulting images were independently analyzed and annotated by two experienced radiologists. Calcified lesions were defined as regions with a CT value greater than 130 Hounsfield units (HU), according to the Expert Consensus on Coronary CT Angiography Scanning and Report Writing (11). The area and maximum CT value of each calcified plaque were recorded, and specific HU coefficients were assigned based on CT value ranges: 1 for 133–199 HU, 2 for 200–299 HU, 3 for 300–399 HU, and 4 for ≥400 HU.

The coronary artery calcium score (CACS) was calculated by multiplying the plaque area by the corresponding HU coefficient for each layer and then summing the calcium scores across all layers. The Agatston scoring algorithm was used to calculate the total CACS based on the CTA findings (12).

**Figure 1:**
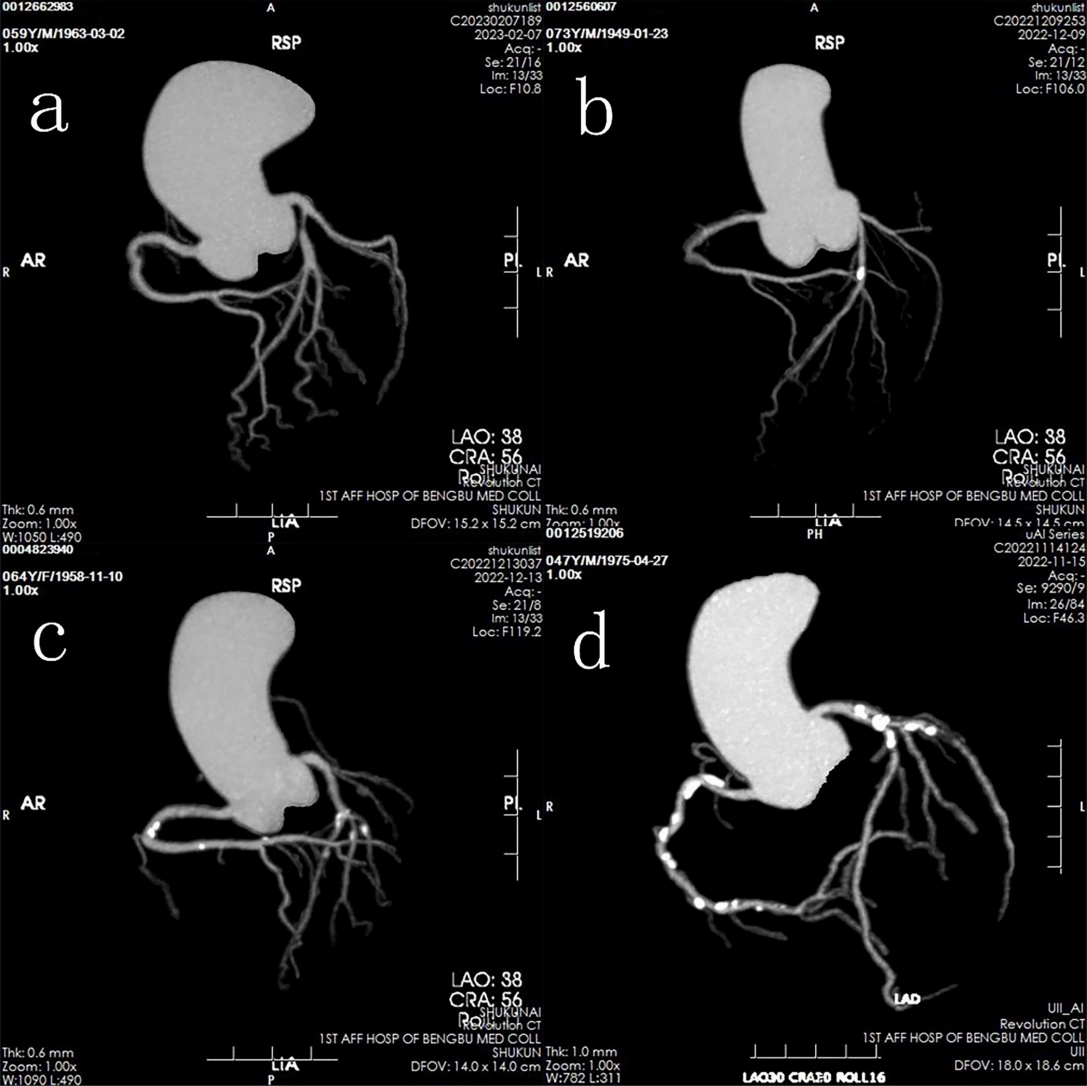
Representative images of four groups based on calcification levels: **(a)** Normal group; **(b)** Mild calcification group; **(c)** Moderate calcification group; **(d)** Severe calcification group.

Patients were classified into two primary groups: a normal group (CACS = 0) and a calcification group (CACS > 0). The calcification group was further subdivided into three categories based on CACS severity: mild calcification (0 < CACS < 100), moderate calcification (100 ≤ CACS < 400), and severe calcification (CACS ≥ 400) (**Figure 1**).

### Statistical analysis

Data analysis was performed using SPSS version 26.0 and R software 4.2.2. Descriptive statistics, including medians and interquartile ranges (IQRs), were used for variables that did not follow a normal distribution, with nonparametric tests applied for group comparisons. Categorical data were presented as frequencies and percentages, and the Chi-square test was used for comparisons between groups. Logistic regression analysis was employed to identify independent risk factors for CAC.

Spearman correlation analysis was conducted to assess the relationships among various indicators. Receiver operating characteristic (ROC) curves were constructed to determine the area under the curve (AUC) and evaluate the predictive value of GRP and IL-1β for CAC. To assess the incremental predictive performance of our models, we employed two reclassification metrics: the Integrated Discrimination Improvement (IDI) and Net Reclassification Index (NRI). Graphical analyses were performed using Graph Pad Prism and Origin software. All hypothesis testing was two-tailed, with statistical significance set at a P value < 0.05.

## Results

### Study population

This study included 120 patients. Patients in the calcification group had a significantly higher prevalence of smoking, diabetes, and hypertension compared to the normal group (p < 0.05). Furthermore, the calcification group exhibited lower serum levels of HDL-C and higher levels of LDL-C and creatinine in comparison to the normal group (p < 0.05). A progressive increase in the prevalence of smoking and hypertension was also observed with increasing calcification severity. No statistically significant differences were found between the normal and calcification groups for other variables (p > 0.05) (**Table 1**).

**Table 1:** Clinical characteristics.

Characteristics	Normal (*n*=44)	Degree of calcification			*H*/χ^2^	*P*
		Mild (*n*=24)	Moderate (*n*=19)	Severe (*n*=33)		
Male [*n* (%)]	20 (45.5)	20 (83.3)	8 (42.2)	19 (57.6)	10.775	0.013
Age/years	58 (52.25, 68.75)	71 (56.25, 79.25)	67 (60.00, 72.00)	(64.50, 76.00)	20.655	0.000
BMI (kg/m^2^)	24.22 (22.31, 26.14)	24.49 (21.24, 27.75)	24.6 (22.66, 28.70)	26.26 (22.75, 28.67)	4.532	0.209
Smoking [n (%)]	3 (6.8)	3 (12.5)	4 (21.1)	12 (46.4)	11.701	0.008
Diabetes [n (%)]	8 (18.2)	7 (29.2)	9 (47.4)	14 (42.4)	7.696	0.053
Hypertension [n (%)]	14 (31.8)	15 (62.5)	12 (63.2)	24 (72.7)	14.721	0.002
TC (mmol/L)	3.81 (3.36, 4.67)	3.60 (3.10, 4.75)	3.26 (2.83, 4.45)	3.82 (3.10, 4.16)	3.241	0.356
TG (mmol/L)	1.04 (0.77, 1.71)	1.23 (0.80, 1.54)	1.14 (0.69, 2.07)	1.12 (0.91, 1.58)	0.746	0.862
HDL-c (mmol/L)	1.17 (1.00, 1.29)	1.00 (0.74, 1.17)	0.91 (0.74, 1.31)	1.02 (0.89, 1.12)	10.745	0.013
LDL-c (mmol/L)	1.97 (1.74, 2.34)	2.72 (2.14, 3.19)	2.15 (1.79, 2.79)	2.71 (2.34, 3.34)	33.264	0.000
LP (a)(mg/L)	137.00 (91.00, 249.00)	187.00 (97.50, 333.25)	68.00 (40.00, 193.00)	256.00 (135.50, 380.00)	10.316	0.016
Ca (mmol/L)	8.92 (8.50, 9.28)	8.56 (8.26, 9.02)	8.88 (8.36, 9.20)	8.96 (8.56, 9.2.)	5.001	0.172
P (mmol/L)	3.58 (3.96, 3.08)	3.32 (2.88, 3.81)	3.50 (3.04, 3.78)	3.35 (2.91, 3.84)	3.618	0.306
Ca-P product	31.78 (34.81, 26.90)	28.65 (24.53, 33.52)	30.35 (27.45, 35.92)	29.95 (26.18, 34.11)	4.799	0.187
ALB (g/L)	42.70 (40.00, 45.45)	42.35 (39.70, 44.83)	42.40 (39.7, 44.60)	43.90 (39.15, 45.50)	0.477	0.924
SCr (μmol/L)	66.50 (55.50, 77.50)	77.00 (67.50, 81.75)	75.00 (62.00, 81.00)	71.00 (66.00, 80.00)	5.338	0.149
CRP (mg/L)	1.15 (0.73, 2.69)	1.20 (0.50, 2.65)	1.20 (00.50, 3.40)	2.10 (1.40, 3.40)	6.719	0.081
ALP (U/L)	60.00 (52.25, 71.75)	59.50 (53.35, 74.00)	65.00 (53.00, 72.00)	73.00 (58.25, 81.50)	5.306	0.151
BUN (mmol/L)	5.28 (4.59, 6.41)	5.39 (4.59, 6.36)	5.00 (4.60, 5.66)	6.61 (4.96, 7.67)	9.19	0.027
Neutrophil	3.04 (2.30, 4.92)	3.33 (2.78, 4.04)	3.43 (2.57, 4.04)	3.90 (3.08, 5.39)	2.939	0.401
Lymphocyte	1.85 (1.32, 2.37)	1.75 (1.23, 2.13)	1.61 (1.26, 2.16)	1.77 (1.32, 2.09)	1.051	0.789
Hemoglobin	130 (119.00, 140.75)	130.50 (113.50, 144.00)	125.00 (115.00, 145.00)	131.00 (119.25, 142.50)	0.059	0.996
PLT	199.00 (158.25, 251.00)	195.00 (163.00, 218.50)	175.00 (131.00, 222.00)	195.50 (161.00, 252.75)	2.526	0.471
NLR	1.82 (1.20, 2.48)	1.86 (1.50, 2.94)	1.82 (1.70, 2.38)	2.25 (1.46, 3.55)	2.143	0.543
PLR	117.83 (80.58, 147.46)	109.03 (88.19, 155.15)	114.93 (86.29, 149.21)	114.04 (86.73, 152.76)	0.81	0.847

BMI, body mass index; TC, testicular cancer; TG, triglyceride; HDL-c, high density lipoprotein cholesterol; LDL-c, Low density lipoprotein cholesterol; LP(a), lipoprotein a; ALB, albumin; SCr, serum creatinine; CRP, C-reactive protein; ALP, alkaline phosphatase; BUN, blood urea nitrogen; PLT, blood platelet; NLR, neutrophil to lymphocyte ratio; PLR, platelet to lymphocyte ratio.

### Serum GRP and IL-1β Levels and coronary artery calcium score

**Figure 2:**
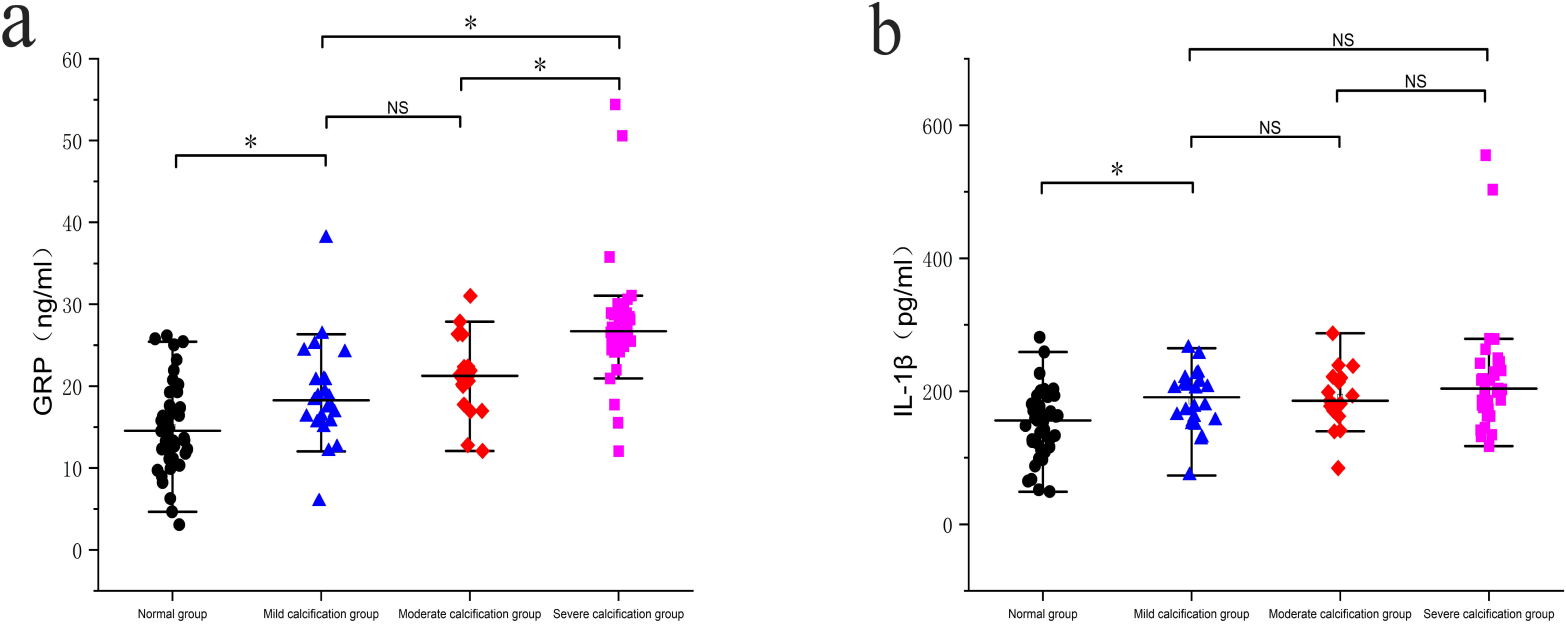
Comparison of serum protein levels in different groups: **(a)** Comparison of serum GRP levels between the groups; **(b)** Comparison of IL-1β levels between the groups. * p < 0.05; NS, No significance.

Serum levels of GRP were significantly higher in the mild, moderate, and severe calcification groups compared to the normal group (p < 0.05). Additionally, the severe calcification group exhibited significantly elevated GRP levels compared to both the mild and moderate calcification groups (p < 0.05). Similarly, serum levels of IL-1β were significantly higher in the mild, moderate, and severe calcification groups compared to the normal group (p < 0.05). However, no statistically significant differences in IL-1β levels were observed between the moderate and severe calcification groups and the mild calcification group (p > 0.05), as shown in **Figures 2a, b**.

### Risk factors for CAC

Multivariate logistic regression analysis identified several independent risk factors for the severity of CAC. These included GRP [odds ratio (OR), 1.202; 95% confidence interval (CI), 1.065–1.356; p = 0.003], IL-1β (OR, 1.011; 95% CI, 1.002–1.020; p = 0.015), age (OR, 1.107; 95% CI, 1.024–1.198; p = 0.011), hypertension (OR, 4.832; 95% CI, 1.082–21.576; p = 0.039), HDL-C (OR, 0.013; 95% CI, 0.001–0.260; p = 0.004), and LDL-C (OR, 13.967; 95% CI, 3.128–62.356; p = 0.001) (**Table 2**).

**Table 2:** Multivariate logistic regression analysis of factors influencing coronary artery calcification (CAC).

Characteristics	B	Wald	OR	95% *CI*	*P*
Age	0.102	6.451	1.107	1.024∼1.198	0.011
Hypertension	1.575	4.257	4.832	1.082∼21.576	0.039
Diabetes	0.867	1.409	2.379	0.569∼9.947	0.235
Smoking	2.416	2.601	11.206	0.594∼211.257	0.107
HDL-c	-4.322	8.099	0.013	0.001∼0.260	0.004
LDL-c	2.637	11.930	13.967	3.128∼62.356	0.001
SCr	0.002	0.010	1.002	0.961∼1.045	0.919
GRP	0.184	8.894	1.202	1.065∼1.356	0.003
IL-1β	0.011	5.859	1.011	1.002∼1.020	0.015

CAC, coronary artery calcification; HDL-c, high density lipoprotein cholesterol; LDL-c, Low density lipoprotein cholesterol; GRP, Gla-rich protein; IL-1β, Interleukin-1β; OR, odds ratio; CI, confidence interval.

### GRP, IL-1β, and their combination in predicting CAC

**Figure 3:**
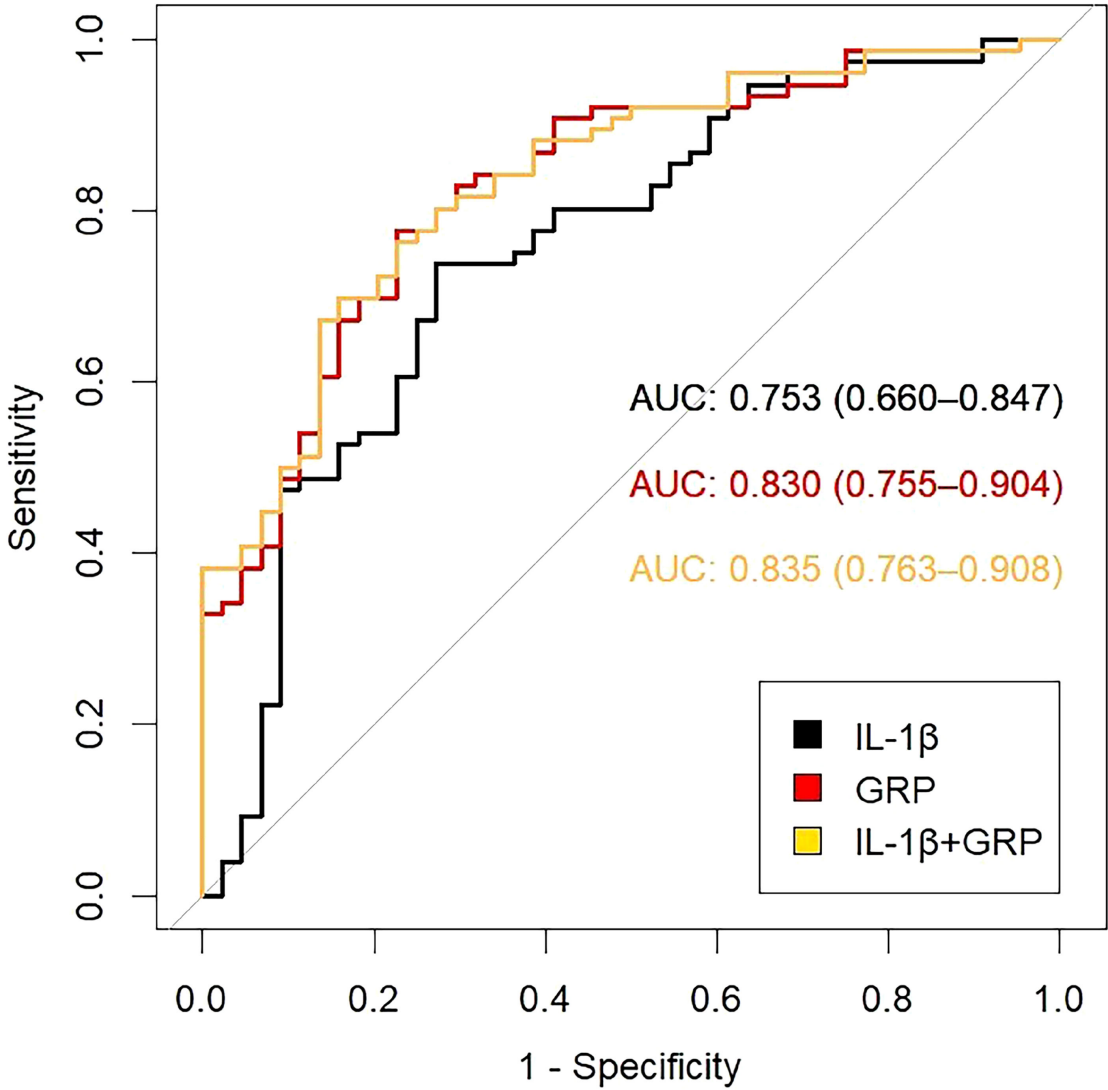
Receiver operating characteristic (ROC) curves for GRP, IL-1β, and their combination in predicting CAC. The area under the curve (AUC) is used to assess the predictive performance of each marker.

ROC curves were generated to assess the diagnostic performance of GRP, IL-1β, and their combination in predicting CAC. The AUC for GRP was 0.830 [95% CI, 0.755–0.904], with an optimal cutoff value of 17.73, resulting in a sensitivity of 77.63% and a specificity of 77.27%. For IL-1β, the AUC was 0.753 [95% CI, 0.660–0.847], with an optimal cutoff value of 170.61, yielding a sensitivity of 73.68% and a specificity of 72.72%. Notably, the combined GRP + IL-1β model achieved the highest discriminatory performance (AUC: 0.835, 95% CI: 0.763-0.908, p < 0.001). While this combined approach demonstrated marginally reduced sensitivity (0.697) compared to individual biomarkers, it conferred markedly enhanced specificity (0.841), suggesting superior capability in correctly identifying CAC. This sensitivity-specificity trade-off is reflected in the Youden index, where the GRP model (0.549) marginally outperformed the combined approach (0.538). The reclassification metrics provide compelling evidence for the incremental value of GRP and the combined model over IL-1β alone. Both the GRP model and the combined GRP+IL-1β model demonstrated statistically significant improvements in NRI (p = 0.003 and p < 0.001, respectively) and IDI (p < 0.001 for both). These metrics quantify the models’ enhanced ability to appropriately reclassify subjects into correct risk categories and improve discrimination between CAC and non-CAC cases (**Table 3**, **Figure 3**).

**Table 3:** Inflammatory biomarkers exhibit significant discriminatory capacity, with their combination yielding superior diagnostic precision.

Model	AUC	SE	*P*	95%CI	Sensitivity	Specificity	Youden index	NRI	Relative IDI
IL-1β	0.753	0.048	<0.001	0.660-0.847	0.737	0.727	0.464	–	–
GRP	0.830	0.038	<0.001	0.755-0.904	0.776	0.773	0.549	0.003	<0.001
GRP+IL-1β	0.835	0.037	<0.001	0.763-0.908	0.697	0.841	0.538	<0.001	<0.001

GRP, Gla-rich protein; IL-1β, Interleukin-1β; AUC, area under the curve; SE, standard error; CI, confidence interval; NRI, Net reclassification index; IDI, Integrated Discrimination Improvement.

### Correlation between GRP, IL-1β and CACS

**Figure 4:**
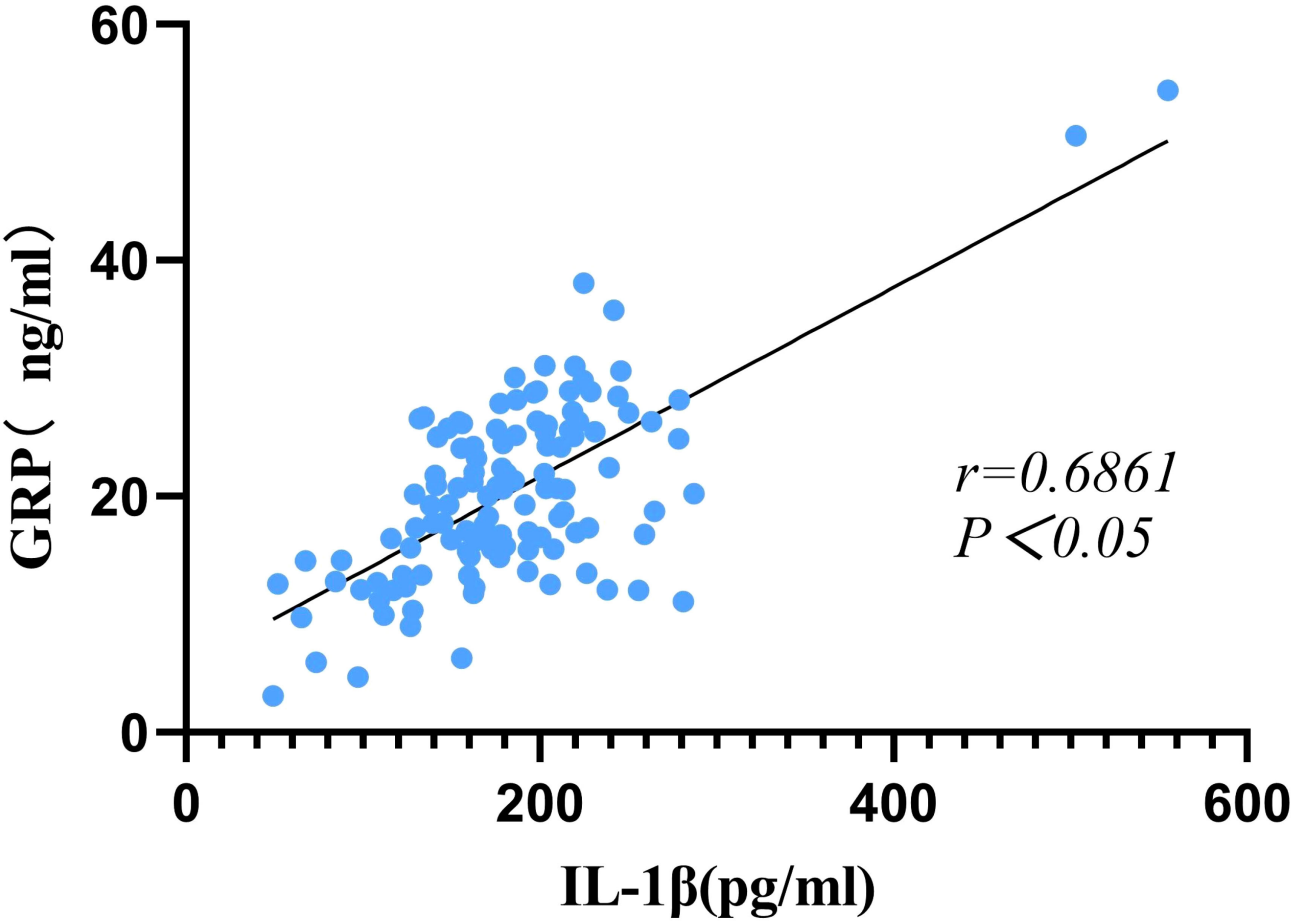
Association between serum GRP and IL-1β levels.

**Figure 5:**
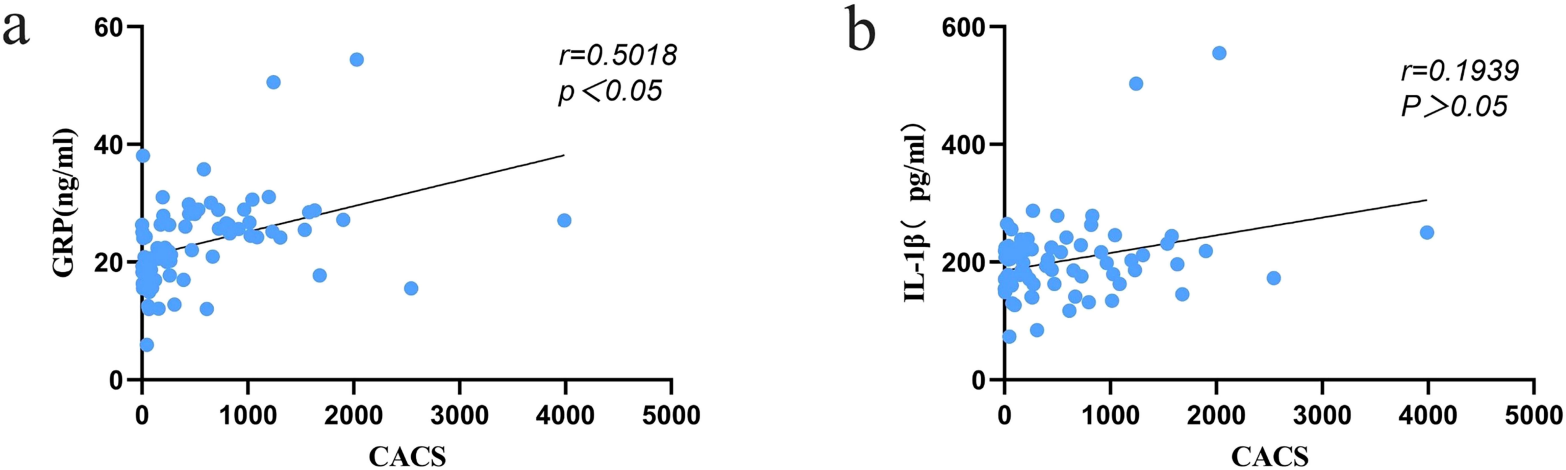
**(a)** Correlation between GRP and CACS; **(b)** Correlation between IL-1β and CACS.

Spearman correlation analysis revealed a statistically significant positive correlation between the serum levels of GRP and IL-1β (r = 0.6861, p < 0.05) (**Figure 4**). Additionally, a significant positive correlation was observed between GRP and the CACS (r = 0.5018, p < 0.05). However, the correlation between IL-1β and CACS was relatively weak and did not reach statistical significance (r = 0.1939, p > 0.05) (**Figure 5**).

## Discussion

In this prospective observational study of patients with suspected CAD, elevated baseline levels of GRP and IL-1β were found to be independently associated with the severity of CAC. These findings suggest that both GRP and IL-1β may provide additional diagnostic value and could serve as useful markers for risk stratification in patients with suspected CAD.

### Inflammatory biomarkers and coronary artery calcification

The current dominant theory regarding the development of atherosclerosis is the “inflammatory hypothesis of atherosclerosis” (13, 14). Previous studies have demonstrated that inflammatory biomarkers may pose a risk similar to conventional cardiovascular risk factors in predicting the incidence and progression of atherosclerotic plaques (15–20). Notably, even with lipid-lowering treatments, inflammation persists as an independent risk factor for cardiovascular diseases (21). Coronary calcification is recognized as a significant manifestation of coronary atherosclerosis, and clinical evidence increasingly suggests that vascular calcification is an independent predictor of myocardial infarction and stroke in the context of atherosclerosis (22, 23). Therefore, identifying alterations in inflammatory biomarkers related to the severity of CAC is essential for developing personalized therapeutic strategies and exploring novel treatment approaches.

### GRP, IL-1β, and coronary artery calcification

GRP, a recently discovered member of the VKDP family, plays a key role in the interaction between inflammation and calcification, particularly in joint tissues affected by osteoarthritis (6, 24, 25). Numerous studies have shown that GRP acts as a calcium chelator and mineral binder within the cardiovascular system, regulating calcium balance and contributing to the calcification of extracellular matrix vesicles originating from vascular smooth muscle cells (26). Silva et al. found a significant correlation between GRP and vascular injury, inflammation, and subsequent inflammatory responses in patients with diabetic nephropathy, emphasizing its role in the development and progression of atherosclerosis (27). Additionally, Viegas et al. identified GRP as a novel molecular mediator in chronic inflammation and calcium-related pathologies. Lipopolysaccharide (LPS) or hydroxyapatite (HA) stimulation upregulated GRP expression in THP-1 monocytes/macrophages, while GRP or GRP-coated calcium phosphate crystals downregulated inflammatory mediators and cytokines, independent of γ-carboxylation. Overexpression of GRP alleviated LPS- and HA-induced inflammation by suppressing TNF-α, IL-1β, and NF-κB signaling, highlighting its potential in modulating inflammation and treating related diseases (28). In our study, we observed a positive correlation between serum GRP levels and CACS, indicating that elevated circulating GRP is associated with increased vascular calcification severity. This suggests that GRP could serve as an early indicator of CAC severity and a valuable tool for monitoring patients with atherosclerosis.

Recent research has highlighted the critical role of IL-1β in the initiation and progression of arterial calcification in atherosclerosis (29–34). Moreover, previous studies have provided strong evidence that IL-1β plays a pivotal role in the pathogenesis of coronary lesions in a mouse model of Kawasaki disease (KD), which can be effectively inhibited by IL-1 receptor antagonists (35). As such, targeting anti-IL-1β therapies could offer a more precise and effective strategy for preventing coronary lesions in KD. Our study similarly found significantly elevated serum IL-1β levels in individuals with CAC compared to those in the control group. IL-1β was identified as an independent risk factor for CAC, suggesting its potential as a standalone predictor. However, the correlation between IL-1β and CACS was modest.

Notably, the combined use of GRP and IL-1β enhanced the predictive capability for determining the presence of CAC. These inflammatory biomarkers provide valuable insights into the underlying pathobiology and may contribute to improved risk stratification in this patient population. The identification of GRP and IL-1β as independent risk factors for CAC underscores the importance of inflammation in the pathogenesis of coronary artery disease. Nevertheless, further studies are needed to validate these findings and determine the significance of monitoring GRP and IL-1β levels in managing inflammation and mitigating excessive inflammatory responses in patients with CAC.

### Limitations

The study’s participant pool was drawn from a single facility, resulting in a relatively small sample size, which may limit the generalizability of the findings to other populations or institutions. Larger-scale studies are needed to validate these results. Additionally, although efforts were made to account for calcification in other areas of the body, potential bias may still exist when using coronary artery calcification as the primary observational indicator.

## Conclusions

The findings indicate a significant association between serum levels of GRP and IL-1β with CAC in patients with CAD, suggesting their potential as independent predictive factors for CAC. Furthermore, the anti-inflammatory and anti-calcification properties of GRP offer promising therapeutic targets for managing CAC.

## Data Availability

The original contributions presented in the study are included in the article/supplementary material. Further inquiries can be directed to the corresponding authors.
